# Supplementary material for: Assessing progress towards Sustainable Development Goal 3.8.2 and determinants of catastrophic health expenditures in Malaysia
Source: PLoS One. 2022 Feb 25;17(2):e0264422. doi: 10.1371/journal.pone.0264422 (PMC8880746; doi:10.1371/journal.pone.0264422)
Supplement: Attachment — Submitted filename: Response to reviewer.docx [file pone.0264422.s001.docx]

Reviewer #1: Title

Page 1; Line 2 – 3: The current title is not appropriate for this article. The title of this article should read: ‘’Assessing catastrophic health expenditure and its associated factors in Malaysia’’ or ‘’Factors associated with catastrophic health expenditure in Malaysia’’.d

# *Thank you for bringing up this idea. The title has been modified to ‘Assessing progress towards Sustainable Development Goals 3.8.2 and determinants of catastrophic health expenditure in Malaysia’*

Page 2: This need to be re-written. Authors should remove SDG 3.8.2.

*Thank you very much for your comment. The aim of the manuscript was to inform readers of the fact that CHE is an SDG 3.8.2 indicator that assesses progress toward UHC 2030. We revised the introduction, but we kept a fresh viewpoint on CHE and SDG 3.8.2 following your recommendation.*

Method

Page 2: This is confusing. The authors claim they applied the WHO method of calculating CHE yet the authors decided to use a threshold of 10% out-of-pocket health spending from total household expenditures to determine CHE.

Under the WHO methodology, CHE occurs when a household’s total out-of-pocket health payments equal or exceed 40% of household’s capacity to pay or non-subsistence spending. Authors should see: Xu (2005)

Reference: Xu K. (2005). Distribution of Health Payments and Catastrophic Health Expenditures: Methodology. Geneva: World Health Organization.

*Thank you for the comment but Xu 2005 is an outdated reference. Please refer to WHO report on tracking UHC: 2017 global monitoring report, Wagstaff et al (2018), on progress on CHS on 133 countries, Cylus et al., (2018) on different CHE calculation method. The budget share method using threshold of 10% and 25% of total consumption are used to determine catastrophic spending specific for SDG 3.8.2*

Results

Page 2: Results are not well presented by the authors. For instance, the statement ‘’the proportion of CHE was 2.8%’’ should read ‘’only 2.8% of households incurred CHE’’.

*Thank you for your suggestion, and the sentence was revised.*

Conclusion

Page 2: Authors should re-write the conclusion to reflect findings from the study.

*There is no need to rewrite since the budget share approach is valid and there was no change in Results, Discussion and Conclusion*

Key words:

Page 2: Authors should consider the following keywords: Catastrophic Health Expenditure, Outof-Pocket Payments, Household Survey, Capacity to pay, Universal health coverage, Malaysia.

*Thank you for your suggestion, some of these keywords were already included, but since there is a limit of five keywords, we had to prioritise SDG as it is a MeSH term (the correct method of formulating keywords)*

Background

Overall remark: Authors need to work on the background section. The current presentation of the background section is not acceptable.

*Thank you for the comment. The introduction was improved but those on* *SDG 3.8.2 was maintained to provide context.*

Page 3 – 6: The background to the study need to be re-written by the authors as it is not coherent.

*Again, I reiterate, the focus of the study is on SDG 3.8.2, the background provided the right context for SDG 3.8.2.*

Page 4; Line 5 – 7: The authors did not provide a ‘’reference’’ for their claim.

*I did. All these sentences were cited from:*

*World Health Organization. Tracking Universal Health Coverage: 2017 Global Monitoring Report. Switzerland: World Health Organization, 2017.*

Page 5; Line 17 – 24 and page 6; 1-24 should be moved to the methods section under a subheading titled ‘’Data Source’’.

*Done.*

The authors need to do a literature review of studies conducted on factors associated with CHE and provide a summary of existing literature in the background section.

*Thank you for your suggestion, we have added a section on factors associated. See last paragraph in the Introduction*

Method

Page 9; Line 8-9: Authors should see Xu 2005 for an explanation of the WHO approach.

*Xu 2005 is an outdated reference. Please refer to a WHO report on tracking UHC: 2017 global monitoring report, Wagstaff et al (2018), on progress on CHS on 133 countries, Cylus et al., (2018) on different CHE calculation method. The budget share method using threshold of 10% and 25% of total consumption are used to determine catastrophic spending specific for SDG 3.8.2*

Page 10: Line 1 – 3: What informed the choice of variables? Authors should cite the relevant literature and theories guiding their selection of variables.

*Thank you for your comment. Relevant literatures are included in the Introduction, see line 95-106.*

Results

Based on the need for a major review of this manuscript, the results of the study is expected to change.

*The results remain the same, as the budget share method using 10% threshold of total consumption is also acceptable in estimating CHE.*

Discussion

 The discussion section is most likely going to change since the authors would have more studies (based on extensive literature review) to compare with the findings from this study.  Authors should also include the implications of their findings for policy under the discussion section.

*The discussion remained the same since the original results were maintained (budget share method using 10% thresholds are also acceptable in estimating CHE).*

Conclusion

Conclusion of this study is also most likely to change.

*The conclusion remained the same since results and discussions were maintained (budget share method using 10% and 25% thresholds are also acceptable in estimating CHE).*

Important note

The authors will benefit from an extensive literature review on this topic and the following studies:

1. Myint CY, Pavlova M, Groot W. (2019). Catastrophic health care in Myanmar: policy implications in leading progress towards universal health coverage. International Journal for Equity in Health. 18(11): 118.

2. Salari P, Giorgio LD, Ilinca S, Chuma J. (2019). The catastrophic and impoverishing effects of out-of-pocket healthcare payments in Kenya, 2018. BMJ Global Health 4:e001809. Doi: 10.1136/bmjgh-2019-001809

3. Nundoochan A, Thorabally Y, Monohur S, Hsu J. (2019). Impact of out of pocket payments on financial risk protection indicators in a setting with no user fees: the case of Mauritius. International Journal for Equity in Health 18:63 https://doi.org/10.1186/s12939-019-09595

4. Proano Falconi D, Bernabe E. (2018). Determinants of catastrophic healthcare expenditure in Peru. Int J Health Econ Manag 18: 425-36.

5. Aregbeshola, B. S., & Khan, S. M. (2018). Determinants of catastrophic health expenditure in Nigeria. European Journal Health https://doi.org/10.1007/s10198-017-0899-1

6. Cleopatra I, Eunice K. (2018). Household Catastrophic Health Expenditure: Evidence from Nigeria. Microeconomics and 10.5923/j.m2economics.20180601.01 Macroeconomics. 6(1): 1-8 DOI:

7. Barasa EW, Maina T, Ravishankar N. (2017). Assessing the impoverishing effects, and factors associated with the incidence of catastrophic health care payments in Kenya. International Journal for Equity in Health 16:31. DOI 10.1186/s12939-017-0526-x

8. Amaya-Lara JL. (2016). Catastrophic expenditure due to out-of-pocket health payments and its determinants in Colombian households. International Journal for Equity in Health. 15:182. DOI 10.1186/s12939-016-0472-z

9. Misra, S., Awasthi, S., Singh, J.V., Agarwal, M., Kumar, V. (2015). Assessing the magnitude, distribution and determinants of catastrophic health expenditure in urban Lucknow, North India. Clin. Epidemiol. Glob. Health 3, 10–16.

10. Buigut, S., Ettarh, R., Amendah, D.D. (2015). Catastrophic health expenditure and its determinants in Kenya slum communities. Int. J. Equity Health 14, 46.

11. Brinda, E.M., Andres, R.A., Enemark, U. (2014). Correlates of out-of-pocket and catastrophic health expenditures in Tanzania: results from a national household survey. BMC Int. Health Hum. Rights 14, 5.

12. Li Y, Wu Q, Xu L, Legge D, Hao Y, Gao L, Ning N, Wan G. (2012). Factors affecting catastrophic health expenditure and impoverishment from medical expenses in China: policy implications of universal health insurance. Bull World Health Organ. 90(9):664–71.

*Thank you for your suggestions. Some of these references were already in the manuscript while others were added in the manuscript. See Reference*

Reviewer #2: I thank the authors for this important study on Financial protection against catastrophic health expenditure in Malaysia. The study gives an updated estimate of catastrophic health expenditures using the 2015/2016 HES survey which is necessary for assessing progress towards achieving SDG 3.8.2 and the factors associated with catastrophic health expenditures to identify the vulnerable groups among the Malaysian population who face CHE. These findings are important for planning and designing health financing policies to ensure financial protection among the vulnerable groups in Malaysia. While this is an important study there are areas that needs to be addressed in the different sections of the paper. The following are my review comments that needs to be addressed to help improve the manuscript:

*I can’t thank you enough for all your constructive comments to improve this manuscript. All revisions were made per your suggestions.*

Abstract

• I suggest the first objective should read “to measure the proportion of households with CHE” since in measuring CHE we are trying to assess progress towards UHC

*Thank you for your comment. Revision made.*

• Sentence number four should be revised to clearly indicate that the authors are using secondary data. Beginning it with “across-sectional study was conducted ….” sounds like the authors conducted the study.

*Thank you for your comment. Revision was made.*

• Sentence number seven needs to be revised to indicate that it is the proportion of sampled households facing CHE and not proportion of CHE

*Thank you for your comment. Revision was made.*

Introduction

• The first Sentence in Paragraph two is not clear it should be revised. One could ask how measuring target 3.8.2 is key to achieving the universal health coverage. Rather it should be clear that measuring target 3.8.3 is key to monitoring progress towards achieving UHC. In the second sentence the last part that reads “until spending on these items is reduced below the level indicated by the poverty line” is unclear it can as well be omitted.

*Thank you for your comment. Revision was made as per your suggestion.*

• The last sentence in paragraph three is unnecessary. What is necessary is for the authors to describe the data which they have used in this study in the methods section

*Thank you for your comment, sentence deleted.*

• In paragraph seven, sentence beginning with “in Malaysia, the data on OOP….” The information could be placed in the methods section. This will help to free up space to describe the health care systems and financing in Malaysia.

*Thank you for your comment. Revision was made as per recommendation.*

• There is need for part of the information in paragraph seven to be transferred into the methods section and include information on the Health care systems and health systems financing in Malaysia. This will help to give the reader background /context on the health systems and how health care is financed in terms of total health expenditures, how much government, private contributes to total health expenditures.

*Thank you for your comment. Revision was made. See line 85-94 in Introduction.*

Methods

• The section on study setting could be described in a paragraph under health systems and health care financing in Malaysia in the introduction section.

*Done. See line 85-94 in Introduction.*

• The first sentence under study design and sample should be revised its unclear

The subheading on Study design and samples was removed.

• The sentence no sampling method was applied is unclear. This could suggest that the 2015/2016 HES study did not use any sampling methods. I suggest that you read the survey report again and try to describe how sampling for the 2015/2016 HES study was performed and put this information under the data source section

*There was no sampling method applied because we included all data that fulfilled the inclusion/exclusion criteria for our data analysis. Although the calculated sample size required (important to give power to the study to detect differences between group) was only 9156, it would be a great waste not to utilize all available data from the HES 2015/2016. Regarding the HES 2015/2016 we did mention the survey applied probability sampling in line 122.*

• The section on study design and study sample can come under data source since it is a secondary analysis the study design and the sample can be described under the data source section. Under the section study design and study sample there is a mention of a PS software; it is unclear what PS stands for.

*The subheading on Study design and samples was removed and its explanations were relocated under Data Source. See 115-131.* *PS stands for Power and Sample Size. The full spelling was inserted in the manuscript, see line 129*

• In the data collection section, the authors may need to provider the recall period for the data on income and all other households ‘expenditures ad may need to indicate whether they are using monthly or annual expenditures

*Since this is secondary data, there was no actual data collection involved. The only data collection was how we obtained and extracted the data from the data custodian. We have indicated that it was monthly consumption in line 119 and the recall period in line 131.*

• In the data collection section, the sentence “The data acquired include household’s characteristics “should be revised to indicate that some of the characteristics are for the household head and others are socioeconomic characteristics of the household.

*Thank you for your comment. I agree, done, see line 146-148*

• Under the data analysis section, the authors did describe how income was classified into low, middle and high income groups. Was the classification based on total household consumption or total household consumption per capita or income? This should be clearly described by the authors. In addition, it is not clear whether the authors are using monthly or annual total household expenditures or income. This has to be clarified.

*We converted monthly household income into three groups based on the cut-off salaries recommended by the Salaries and Wages Survey Report (see line 173-174). We used monthly consumption (line 149-150) and monthly income (line 173)*

• When defining how CHE was measured it should be made clear that both income and total household expenditure were used since the results section presents the proportion of households that faced CHE based on both income and household expenditures.

*We have decided to use just one indicator and one threshold, therefore we have removed Table 2*

Results

• The first sentence in the results section is unnecessary as the sample size has already been given in the methods section. This sentence should be replaced with a sentence that makes reference to the results in table 1 before the results are presented.

*We retained this sentence to show readers that although the required sample size was 9156, but the overall data included in the analysis was 13015 (line 187-189). We also wanted to highlight the proportion of households that did not report any health spending. A sentence that introduced Table 1 was added to the paragraph (line 190)*

• The reported mean age of the household’s head on page 10 does not match with what has been reported in Table1 please check this.

*Thank you for your attention to detail. Correction was made*

• I suggest reporting the results using Malaysian currency (MYR) and indicating under the table of results that for the year of the survey 1MYR=0.24USD

*I agree. Thank you. Corrections made.*

• The heading for the section on measurement of proportion of households facing CHE should be revised as “Measurement of the proportion of households with CHE”

*I combine two subheading that reads “Proportion of households with CHE and its associated factors”*

• As mentioned in point number one you need to make reference to table 2 before presenting the results ;this makes it easier for the reader to follow ie “Table 2 gives of the proportion of households facing CHE….”

*No longer relevant as Table 2 was removed.*

• The last sentence under the section measurement of the proportion of households with CHE is unclear it seems there is something missing. It should be revised.

*No longer relevant as Table 2 was removed.*

• The second and third sentences under section on Factors associated with CHE are unnecessary and can be omitted as no results on multicollinearity between the variables has been presented.

*Done, these sentences were removed*

• The heading for table 3 should be revised to read “Association between socio-demographic characteristics of households and CHE using multiple logistic regression n=13015”

*Done. The title for Table 3 was changed as suggested.*

Discussion

• The first sentence could be improved to read “ ……,this study revealed that 2.8% of Malaysian households incurred CHE.

*Done.*

• One of the important/main finding of this study is that the proportion of households that incurred CHE increased in 2015/2016 compared to the estimates in 2004/2008 and 1998/1999; the authors have briefly discussed why the estimate of CHE may have increased, however the implications of this increase has not been discussed. The implications of the increase in CHE needs to be discussed.

*Done. See line 244-247.*

• Sentence number 6 in paragraph 1 which begins with “however, the proportion of CHE …..” this should begin as a separate paragraph since the information is comparing CHE estimates in Malaysia and globally while the rest of the information in paragraph 1 is comparing estimate of CHE using 2015/2016 HES to 2004/2008 and 1998/1999 HES

*Done*

• In paragraph 4 in the second sentence the authors have cited the 2015 National Health Morbidity survey report but no reference has been provided; please provide the reference wherever this report has been cited i.e. in paragraph 5 as well. The use of urbanites in paragraph 4 should be revised and a better word that describe urban residents should be used.

*The reference was located at the last sentence in the paragraph (line 286). The word urbanite was changed to urban populations and households in urban areas.*

• In paragraph 7 the authors have cited the salaries and wages survey report and no reference has been provided.

*Thank you for noting this flaw. The reference from the Department of Statistics, 2017 was provided at the end of the sentence. See line 334.*

• In paragraph 8 the authors provide an important finding of the study in which rural households are more likely to face CHE than urban households. Although the authors have described why this may be so; they have not discussed the policy implications of this finding. The implications of these findings should be discussed.

Conclusion

• In sentence 1 “The SDG 3.8.2” should be omitted and the sentence should be revised as previously suggested.

*Term omitted.*

• There is need to rearrange the sentences to make it easier for the readers to follow for example sentence number 3 in this paragraph should come after sentence one. In addition, the use of despite in sentence number 3 is unnecessary and it can be omitted.

*Thank you. Correction was done based on the reviewer’s recommendation.*

• The last sentence beginning with “A health policy in Malaysia ……..” is very important but it is unclear. It should be revised to indicate the kind of policy and to make it easy for the reader to understand

*We have improved on the sentence. See line 401-402.*

Minor comments

• There are a number of grammatical errors in the discussion; the paper could benefit a lot from English editorial services. This will make the discuss section easy to follow.

*We tried our best as non-native English speakers and since we cannot afford English editorial service, we used Quill Bot to improve on the sentence structures and grammatical errors.*

• Authors should also check the use tenses in the paper.

*Done*

• When resubmitting please use line numbers for easy reference when reviewing the manuscript.

*Done, line numbers were inserted.*

[NOTE: If reviewer comments were submitted as an attachment file, they will be attached to this email and accessible via the submission site. Please log into your account, locate the manuscript record, and check for the action link "View Attachments". If this link does not appear, there are no attachment files.]

While revising your submission, please upload your figure files to the Preflight Analysis and Conversion Engine (PACE) digital diagnostic tool, https://pacev2.apexcovantage.com/. PACE helps ensure that figures meet PLOS requirements. To use PACE, you must first register as a user. Registration is free. Then, login and navigate to the UPLOAD tab, where you will find detailed instructions on how to use the tool. If you encounter any issues or have any questions when using PACE, please email PLOS at figures@plos.org. Please note that Supporting Information files do not need this step.
