# Supplementary material for: Assessing progress towards Sustainable Development Goal 3.8.2 and determinants of catastrophic health expenditures in Malaysia
Source: PLoS One. 2022 Feb 25;17(2):e0264422. doi: 10.1371/journal.pone.0264422 (PMC8880746; doi:10.1371/journal.pone.0264422)
Supplement: Attachment — Submitted filename: Response to reviewer.docx [file pone.0264422.s002.docx]

Response to reviewer

There was only one comment as listed below.

Reviewers' comments:

Reviewer #2: The authors have addressed all the comments accordingly. The manuscript is ready for publication. However, i suggest that the authors should go through the manuscript again to check for grammatical errors. For example please check and revise line191,lines 62-62,line 64 and lines 65-66

*Response: The whole manuscript has been English edited and proofread using Quillbot and Grammarly. All grammar errors have been corrected and sentences were restructured.*
